# A Case Report of Acute Intermittent Porphyria Accompanied by Severe Peripheral Neuropathy

**DOI:** 10.3390/diagnostics16121809

**Published:** 2026-06-11

**Authors:** Yanting Liu, Jian Cao, Fei Han, Qianlong Chen, Hui You, Huadong Zhu, Yi Li, Anlei Liu, Jing Yang

**Affiliations:** 1 State Key Laboratory of Complex Severe and Rare Diseases, Critical and Emergency Pharmaceuticals & Medical Devices Innovation Lab, Department of Emergency, Peking Union Medical College Hospital, Chinese Academy of Medical Sciences and Peking Union Medical College, No. 1 Shuaifuyuan, Wangfujing, Dongcheng District, Beijing 100730, China; 2 Department of Radiology, Peking Union Medical College Hospital, Chinese Academy of Medical Sciences and Peking Union Medical College, No. 1 Shuaifuyuan, Wangfujing, Dongcheng District, Beijing 100730, China; 3 Department of Neurology, Peking Union Medical College Hospital, Chinese Academy of Medical Sciences and Peking Union Medical College, No. 1 Shuaifuyuan, Wangfujing, Dongcheng District, Beijing 100730, China; 4 State Key Laboratory of Cardiovascular Disease, Beijing Key Laboratory for Molecular Diagnostics of Cardiovascular Diseases, Diagnostic Laboratory Service, Fuwai Hospital, National Center for Cardiovascular Diseases, Chinese Academy of Medical Sciences and Peking Union Medical College, No. 167, Beilishi Road, Xicheng District, Beijing 100037, China

**Keywords:** acute intermittent porphyria, abdominal pain, peripheral neuropathy, *HMBS* mutation

## Abstract

**Background:** Acute intermittent porphyria (AIP) is the most common and severe form of acute hepatic porphyria, caused by heterozygous mutations in the *HMBS* gene. Due to its non-specific clinical manifestations and low clinical awareness among clinicians, AIP is frequently misdiagnosed, leading to significant diagnostic delays and potentially fatal complications. **Case presentation:** We report a 20-year-old female patient who presented with a 9-month history of recurrent abdominal pain, paralytic ileus, unexplained liver injury, and hyponatremia, followed by progressive limb weakness. She was initially misdiagnosed with Guillain–Barré syndrome (GBS) and received intravenous immunoglobulin and systemic glucocorticoids. However, her condition deteriorated, and she developed life-threatening respiratory muscle paralysis requiring invasive mechanical ventilation. The diagnosis of AIP was confirmed by positive urinary porphobilinogen (PBG) testing and identification of the heterozygous *HMBS* c.517C>T pathogenic variant. The patient was treated with high-dose carbohydrate loading therapy and comprehensive supportive care, resulting in gradual clinical improvement. **Discussion and Conclusions:** This case exemplifies the substantial diagnostic challenges associated with AIP, especially when it manifests with peripheral neuropathy that closely mimics GBS. The triad of absent albuminocytologic dissociation in cerebrospinal fluid, preceding visceral symptoms, and inadequate response to standard first-line GBS therapy should immediately raise clinical suspicion for AIP. Enhanced clinical awareness of this rare disorder and timely implementation of urinary PBG screening are of paramount importance to prevent irreversible neurological complications and optimize long-term patient outcomes.

## 1. Background

Porphyria refers to a group of metabolic disorders caused by abnormal enzyme activities during heme biosynthesis, which are clinically categorized into acute and non-acute (also known as cutaneous) porphyrias [1]. The four types of acute porphyrias, all occurring in the liver, are collectively known as the acute hepatic porphyrias (AHP) [2]. Acute intermittent porphyria (AIP), the most common form of AHP, is caused by mutations in the gene encoding hydroxymethylbilane synthase (*HMBS*), the third key enzyme in the heme biosynthesis pathway [3]. *HMBS* deficiency leads to the accumulation of toxic porphyrin precursors, predominantly δ-aminolevulinic acid (ALA) and porphobilinogen (PBG), causing dysfunction of the peripheral, central, and autonomic nervous systems [4]. The vast majority of pathogenic *HMBS* mutation carriers remain asymptomatic throughout their lifetimes, with the actual prevalence of clinically overt AIP in the Chinese population estimated at 1 in 1059 [5]. AIP is an autosomal dominant disorder with very low penetrance (0.5–1.0%) and high heterogeneity [6]. Peripheral neuropathy develops in up to 40% of patients with AIP during acute attacks and may progress to life-threatening paralysis in severe cases [7]. Due to its non-specific clinical presentation and the lack of a positive family history in most cases, AIP is frequently underrecognized or misdiagnosed, posing substantial challenges to timely diagnosis and clinical intervention.

Herein, we report the case of a female patient with AIP who initially presented with abdominal pain, paralytic ileus, liver injury, and hyponatremia, followed by the onset of progressive muscle weakness. The patient was misdiagnosed with Guillain–Barré syndrome (GBS), and received intravenous immunoglobulin (IVIG) and glucocorticoids before the definitive diagnosis. Due to respiratory failure caused by respiratory muscle involvement, the patient underwent tracheal intubation and mechanical ventilation. This case report aims to raise clinical awareness of this rare disorder among clinicians and to facilitate timely recognition and accurate diagnosis to improve patient outcomes.

## 2. Case Presentation

### 2.1. Patient Information and Clinical Course Prior to Admission

A 20-year-old woman was admitted to our hospital on 29 May 2025, with a 9-month history of intermittent abdominal pain and a 3-month history of limb weakness, and an unintentional weight loss of 15 kg over the disease course. The patient had no remarkable past medical history or family history of hereditary diseases.

In July 2024, the patient developed persistent epigastric distending pain, nausea, anorexia, and obstipation a few days after an upper respiratory tract infection. Non-contrast abdominal computed tomography (CT) revealed intestinal obstruction, and her symptoms resolved completely after conservative symptomatic treatment. Between November 2024 and February 2025, the patient experienced recurrent abdominal pain and bloating without identifiable triggers, accompanied by nausea and vomiting. Biochemical tests showed elevated alanine aminotransferase (ALT, 136 U/L, reference range: 7–40 U/L) and bilirubin (total 50.9 μmol/L, reference range: 5.1–22.2 μmol/L; direct 12.4 μmol/L, reference range: ≤6.8 μmol/L), severe hyponatremia (nadir serum sodium 127 mmol/L, reference range: 135–145 mmol/L), hypokalemia, hypomagnesemia, and hyperlipidemia. Wilson disease and autoimmune liver disease were excluded based on normal ceruloplasmin, negative antinuclear antibodies and negative autoimmune liver disease-associated antibody panels. Imaging showed colonic dilatation, while colonoscopy revealed no organic stenosis or mucosal lesions. Her gastrointestinal symptoms were alleviated with symptomatic treatment, and liver function parameters improved temporarily after hepatoprotective therapy; however, the specific medications were not recorded in the original medical records from the external hospital. During this period, she also experienced transient hand tremors and mild wrist drop, which resolved spontaneously within 3 days without specific intervention.

In April 2025, the patient had another episode of abdominal pain, followed by difficulty raising her upper limbs, severe bilateral wrist drop, squatting weakness, and gradually worsening weakness in both lower limbs with decreased exercise tolerance. Cerebrospinal fluid (CSF) examination showed no abnormal findings in routine, biochemical or pathogenic tests. No significant abnormalities were found on magnetic resonance imaging (MRI) of the head, cervical and thoracic spine, and abdomen. Electromyography (EMG) showed neurogenic damage (cervical, thoracic and lumbar segments), and sympathetic skin response was absent in the bilateral upper and lower extremities. Therefore, GBS was suspected, and the patient was treated with IVIG (400 mg/kg/day for 5 days), neurotrophic agents, hepatoprotective therapy and gastrointestinal decompression. Details of the neurotrophic and hepatoprotective pharmacotherapy were not documented in the transferred external hospital records. The muscle strength of lower limbs improved temporarily, allowing her to walk up to 300 m independently, but her upper limb symptoms remained unrelieved, and recurrent abdominal pain persisted. In May 2025, methylprednisolone was administered (40 mg twice daily for 3 days), with no observable improvement in clinical symptoms. Given the patient’s 9-month history of recurrent abdominal pain, paralytic ileus, liver injury, and hyponatremia, along with a poor response to IVIG and methylprednisolone therapy for suspected GBS, the physicians at the referring hospital began to suspect a diagnosis of AIP. They performed a urine PBG-sunlight test and observed that the urine color darkened significantly after 2 h of sunlight exposure, which highly indicated AIP. The patient was then transferred to our hospital.

### 2.2. Physical Examination on Admission

The physical examination upon this admission revealed decreased muscle tone in all four limbs, with muscle strength graded 0/5 in both upper and lower extremities. Tendon reflexes were absent in all extremities. Sensory system examination was normal. Full extraocular movements were intact, with horizontal nystagmus noted. The patient had a weak voice, appeared listless, responded appropriately to questions, and had normal orientation. Physical examination also showed poor somatic development and malnutrition, accompanied by a mild anemic appearance, and icteric skin and mucosa. The bowel sounds were significantly diminished. Sinus tachycardia (107 beats per minute) was observed on electrocardiogram.

### 2.3. Diagnostic Evaluation and Confirmation

After admission, laboratory tests showed positive urinary PBG, along with mild anemia (hemoglobin [HGB] 104 g/L, reference range: 110–150 g/L), severe liver injury (ALT 568 U/L, reference range: 7–40 U/L), cholestatic jaundice (total bilirubin 96.9 μmol/L, reference range: 5.1–22.2 μmol/L; direct bilirubin 73.1 μmol/L, reference range: ≤6.8 μmol/L), and critical electrolyte disorders (serum sodium 129 mmol/L, reference range: 135–145 mmol/L; serum potassium 2.8 mmol/L, reference range: 3.5–5.5 mmol/L). Non-contrast abdominopelvic CT revealed extensive intestinal pneumatosis, suggestive of paralytic ileus. A segment of markedly dilated small bowel loops was noted in the left upper quadrant, lateral to the splenic flexure of the colon (Figure 1). A presumptive diagnosis of AIP was therefore established. On 31 May 2025, the patient developed acute dyspnea, with room-air SpO2 acutely dropping to 73%. Noninvasive respiratory support failed to improve oxygenation significantly. Given respiratory failure secondary to AIP-related respiratory muscle paralysis, the patient underwent emergency endotracheal intubation with initiation of invasive mechanical ventilation, followed by immediate transfer to the emergency intensive care unit (EICU). Genetic analysis revealed that the patient carried a heterozygous missense variant in the *HMBS* gene (c.517C>T, p.Arg173Trp), which was inherited from her mother and was not present in her father or elder brother (Figure 2). A definitive diagnosis of AIP was confirmed. Blood and urine heavy metal toxicity screening was negative. Brain MRI with susceptibility-weighted imaging (SWI) showed no significant abnormalities. Repeat EMG confirmed peripheral neurogenic damage in all four extremities. Table 1 shows that bilateral median, ulnar and peroneal motor nerves failed to elicit any measurable compound muscle action potentials (CMAPs). For sensory nerves, the bilateral median and left ulnar nerves showed significantly reduced sensory nerve action potential (SNAP) amplitudes, while conduction velocities remained within normal limits. Bilateral tibial nerve F-wave studies demonstrated significantly prolonged mean latencies with normal 100% response rates (reference range: ≥80%) (Figure 3). F-wave conduction velocities (FCV) were not measured. The waveforms were clear and consistent without dispersion or absence (Figure 3).

### 2.4. Treatment, Outcome and Follow-Up

Regarding treatment, because hemin, the first-line treatment for acute attacks of AIP [6], was unavailable in our hospital, high-dose carbohydrate loading therapy was initiated, with a total daily intravenous glucose intake exceeding 300 g. Given persistent respiratory muscle weakness, vocal cord dysfunction, and dysphagia, prolonged neurological recovery was anticipated. The patient therefore underwent tracheotomy on 5 June. She was gradually weaned to high-flow nasal cannula oxygen therapy. Concurrently, comprehensive interventions were implemented, including gastrointestinal decompression, enema, acid suppression, prokinetic agents, choleretic therapy, electrolyte correction, neurotrophic support, heart rate stabilization, and anti-infective therapy. Fluid restriction and sodium supplementation were given to correct hyponatremia caused by the syndrome of inappropriate antidiuretic hormone secretion (SIADH). Mirtazapine was prescribed to manage anxiety and depression secondary to the patient’s severe somatic illness. On 13 June, IVIG (20 g once daily for 3 days) was administered to facilitate neurological recovery. In addition, the patient received structured rehabilitation interventions, including swallowing and phonation training, neuromuscular electrical stimulation, and muscle strength training for all extremities. Additionally, the patient was diagnosed with hypogonadotropic hypoestrogenism, for which a drug-induced etiology could not be definitively ruled out. Menopause treatment was scheduled for re-evaluation upon functional recovery of the hypothalamic-pituitary-gonadal axis.

Following treatment, the patient’s abdominal distension resolved, with no recurrence of abdominal pain and a gradual return of normal bowel function. Liver function tests normalized gradually, and electrolyte imbalances were fully corrected. Combined oral and nasogastric glucose feeding was established. At discharge, her limb muscle strength had improved as follows: proximal lower limbs grade 3-/5, distal lower limbs grade 1–2/5, proximal upper limbs grade 2/5, and distal upper limbs grade 1/5. The patient was transferred to a local hospital for ongoing rehabilitation on 14 July 2025. She received a 6-month course of medical menopause therapy starting in August 2025 and has remained amenorrheic to date. In October 2025, she underwent successful tracheostomy decannulation, and in November 2025, she began attempting to stand and walk with assistance. At the most recent follow-up in May 2026, the patient has remained free of recurrent acute abdominal pain since hospital discharge. She has achieved near-complete independence in all basic activities of daily living, including dressing, grooming, and feeding. The tracheostomy stoma has completely healed, with no vocal cord dysfunction, dysphagia, or respiratory impairment. She can now ambulate independently for 50–60 m on level ground, but still has difficulty performing squatting and standing maneuvers. Follow-up electrophysiological studies have not been conducted. A full neurophysiological reassessment is scheduled for the second half of 2026.

## 3. Discussion

AIP is a rare disease caused by heterozygous mutations in the gene encoding *HMBS* [1]. Nevertheless, the vast majority of pathogenic *HMBS* mutation carriers remain asymptomatic. AIP is characterized by heterogeneous neurovisceral episodes and multisystem involvement, manifesting as abdominal pain, vomiting, muscle weakness, psychiatric disturbances, respiratory failure, epilepsy, and tachycardia [6], frequently leading to diagnostic delay and inappropriate management. Studies have demonstrated that the median interval from symptom onset to definitive AIP diagnosis ranges from 1 to 17 years, with a misdiagnosis rate exceeding 70% [8,9]. Herein, we report a severe case of AIP in a 20-year-old female patient who presented with a 9-month history of recurrent abdominal pain, progressive liver injury, and severe peripheral neuropathy, and was initially misdiagnosed with GBS before a definitive diagnosis was established. This case highlights the key clinical pitfalls in the diagnosis and management of AIP, and provides clinical experience in the treatment of severe AIP in settings where hemin is unavailable.

The diagnosis of AIP remains a major clinical challenge worldwide. This patient’s early non-specific gastrointestinal manifestations made it difficult to differentiate the condition from common gastrointestinal disorders. The subsequent misdiagnosis of GBS was clinically understandable, given the progressive symmetric limb weakness and electromyographic evidence of neurogenic damage. However, misdiagnosis of GBS led to the glucocorticoid administration, a known trigger of acute AIP attacks that may exacerbate the patient’s condition [10]. Importantly, several clinical red flags should have raised suspicion for AIP earlier in the disease course, and these features also define the key differential diagnostic points between AIP and GBS. AIP and GBS share multiple clinical overlaps. GBS is an immune-mediated acute inflammatory peripheral neuropathy, typically presenting with post-infectious symmetric ascending paralysis without prominent preceding visceral symptoms, and albuminocytologic dissociation of CSF analysis is the hallmark feature [11,12]. However, prior to the onset of limb weakness, this patient had a long history of recurrent abdominal pain, paralytic ileus, and liver injury. CSF analysis showed no significant abnormalities, and she exhibited only a minimal transient response to IVIG, the first-line treatment for GBS [13]. The diagnostic breakthrough came when the attending physicians at the referring hospital recognized these atypical features and initiated the urine PBG-sunlight test for AIP. After admission to our hospital, qualitative urinary PBG testing was positive. According to the clinical practice guidelines for porphyrias [14], a positive urinary PBG test establishes the clinical diagnosis of AHP. Genetic testing was subsequently performed to identify the specific pathogenic variant in the *HMBS* gene, confirm the subtype diagnosis of AIP, and facilitate genetic counseling and screening for the patient’s at-risk family members. Therefore, this case highlights the critical importance of early differential diagnosis between AIP and GBS. AIP must be routinely excluded via timely urinary PBG screening in all patients with suspected GBS accompanied by unexplained abdominal pain, multisystem involvement, or poor treatment response.

This patient developed severe, rapidly progressive peripheral neuropathy, manifesting as flaccid quadriplegia, dysphagia, and respiratory muscle paralysis. Peripheral neuropathy occurs in 10–40% of AIP cases [15], usually developing after visceral symptoms, with 80% of patients showing predominant proximal muscle weakness that may progress to tetraplegia [16]. Respiratory muscle involvement can occur in up to 50% of untreated patients [16] and may become life-threatening. The EMG findings in our case revealed predominant motor axonal damage, consistent with the typical neuropathological feature of porphyric neuropathy [17]. Sensory neuropathy can manifest as limb pain, glove-and-stocking or patchy numbness, and other sensory disturbances [16]. No signs of central nervous system involvement, such as seizures or consciousness disturbance [18], were observed in this case. Hyponatremia in AIP is primarily caused by SIADH, resulting from hypothalamic-pituitary neurotoxicity induced by accumulated ALA and PBG [19]. Classic autonomic manifestations of AIP in this patient included abdominal pain, abdominal distention, paralytic ileus, nausea, vomiting, and sinus tachycardia, secondary to splanchnic autonomic dysfunction. It is acknowledged that heme deficiency reduces the activity of rate-limiting enzymes involved in the hepatic tryptophan degradation pathway, resulting in tryptophan accumulation and enhanced 5-hydroxytryptamine (5-HT) synthesis [20]. Excessive 5-HT drives extensive autonomic dysfunction [20]. Elevation of liver enzymes and bilirubin was attributed to direct hepatotoxicity of ALA and PBG via oxidative stress and hepatocyte apoptosis, alongside disrupted bile metabolism and a vicious cycle of hypoperfusion, metabolic disturbance, and inflammation-mediated hepatic damage [8,21,22]. Other etiologies of acute liver injury were ruled out, supporting AIP-related hepatic impairment. As for the psychiatric manifestations, the patient’s anxiety and depression were considered adjustment-related issues secondary to physical illness.

More than 500 pathogenic variants of the *HMBS* gene have been identified. In this patient, genetic testing identified a heterozygous missense variant c.517C>T in the *HMBS* gene, which is the most prevalent pathogenic variant in Chinese AIP patients, and a well-recognized high-frequency mutation worldwide [23,24]. This mutation causes a severe loss of HMBS enzyme activity by disrupting the enzyme’s active center [25]. Currently, there is no conclusive evidence supporting a genotype–phenotype correlation in AIP. Patients harboring the *HMBS* c.517C>T variant display a wide spectrum of clinical severity. This case exhibited progressive limb weakness, dysphagia and severe respiratory muscle paralysis, while her mother, a carrier of the identical *HMBS* mutation, remained asymptomatic. This variation indicates that other genetic and/or environmental factors contribute to AIP attacks. Certain triggering factors, such as medications, fluctuations in sex hormones, calorie or carbohydrate restriction, alcohol, tobacco, and stress, can precipitate AIP attacks via the accumulation of toxic upstream heme intermediates [6], though in this case, no obvious triggering factors were identified.

The core therapeutic goal for acute AIP attacks is to inhibit the activity of hepatic δ-aminolevulinic acid synthase 1 (ALAS1)—the first and rate-limiting enzyme in the heme biosynthetic pathway—thereby reducing the accumulation of ALA and PBG [26]. Intravenous hemin is the first-line treatment, while carbohydrate loading therapy (300–400 g/d), administered either orally or via intravenous glucose infusion, serves as an alternative treatment in hemin-unavailable regions [26]. Glucose can stimulate insulin secretion and downregulate peroxisome proliferator-activated receptor gamma coactivator 1-α (PGC-1α), thereby suppressing hepatic ALAS1 and reducing ALA synthesis [27,28]. Identification and prompt discontinuation of all potential triggering factors, and rational use of porphyria-safe medications for symptomatic and supportive management are also required to avoid exacerbating the acute attack.

Neurological recovery is a long and slow process. High-dose carbohydrate loading effectively halts acute AIP attacks but exerts little effect on the repair of injured peripheral nerves. However, the resolution of acute AIP attacks and supportive care can facilitate a favorable physiological environment for nerve repair. The second course of IVIG was administered empirically. Although IVIG has no conclusive evidence supporting its efficacy for AIP-related neuropathy, we considered that its immunomodulatory and anti-inflammatory properties might potentially alleviate secondary neuroinflammation and indirectly promote neurological functional recovery. Longer follow-up and neurophysiological reassessment are needed. For long-term neurological recovery, givosiran, a double-stranded small interfering ribonucleic acid (siRNA) that targets hepatic ALAS1 mRNA and thereby reduces levels of ALA and PBG, has emerged as the most effective therapeutic agent currently [29]. Givosiran has been approved in the United States for the treatment of acute hepatic porphyria and in Europe for the prevention of recurrent attacks [29]. Emerging real-world evidence indicates that givosiran can facilitate substantial neurological improvement even in patients with severe AIP-related tetraplegia [30,31]. However, the prohibitive cost of givosiran remains a significant barrier to its global accessibility. In our case, givosiran was not available at our institution during the patient’s acute presentation, and treatment was therefore restricted to high-dose carbohydrate loading and comprehensive supportive care. Early initiation of givosiran therapy in similar cases may lead to more rapid and complete neurological recovery.

## 4. Conclusions

This case illustrates the significant diagnostic challenges associated with AIP presenting as severe progressive quadriplegia and respiratory failure, a clinical phenotype that is frequently misdiagnosed as GBS. AIP should be routinely considered in the differential diagnosis of all patients presenting with unexplained recurrent abdominal pain, especially when accompanied by multisystem involvement such as peripheral neuropathy, liver injury and electrolyte abnormalities (particularly hyponatremia). In addition, in patients with suspected GBS who exhibit preceding visceral symptoms, normal CSF findings, or a poor response to standard first-line immunotherapy, timely urinary PBG screening is essential to exclude AIP. Moreover, in settings where intravenous hemin and givosiran are unavailable, high-dose carbohydrate loading therapy combined with comprehensive supportive care can effectively stabilize patients and facilitate significant long-term neurological recovery. A limitation of this report is the absence of repeated nerve conduction studies to correlate clinical improvement with electrophysiological recovery.

## Figures and Tables

**Figure 1:**
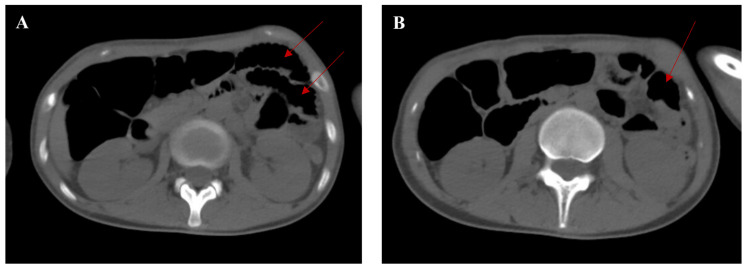
Non-contrast abdominopelvic computed tomography (CT) findings of the patient (on 29 May 2025). (**A**) The CT image shows dilated small bowel loops (red arrows) in the left upper quadrant, lateral to the splenic flexure of the colon. (**B**) The CT image at a lower level shows the dilated small bowel loop (red arrow).

**Figure 2:**
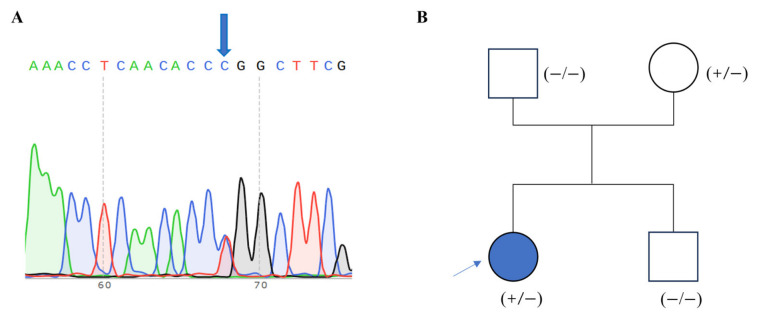
Peripheral blood genetic sequencing results and pedigree chart of the patient with acute intermittent porphyria (AIP). (**A**) The genetic sequencing result showed a heterozygous missense variant in the *HMBS* gene (c.517C>T). The blue arrow marks the exact position of the nucleotide substitution. (**B**) The *HMBS* c.517C>T variant was identified in the proband and her mother. The blue arrow indicates the proband.

**Figure 3:**
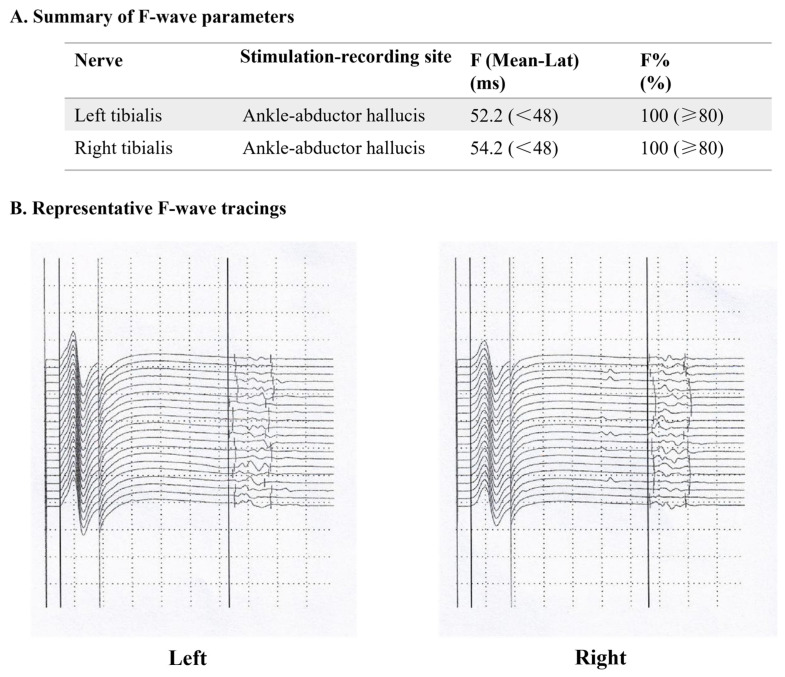
F-wave study results of bilateral tibial nerves show markedly prolonged mean latencies with preserved 100% response rates. (**A**) Summary of F-waves parameters. Values are presented as measured values (normal reference range). All reference ranges are from the neurophysiology laboratory of our institution. (**B**) Tracings of F-waves. Calibration: 5 mV/division (vertical), 8 ms/division (horizontal). (**Left**), left tibial nerve; (**Right**), right tibial nerve. For each subplot, the right vertical line marks the minimum F-wave latency.

**Table 1:** Nerve conduction study findings in this case (13 June 2025).

Nerve	Stimulation-Recording Site	Latency (ms)	Amplitude	Conduction Velocity (m/s)	Distance (mm)
Motor nerve					
Bilateral Median	Wrist-APB	NR	NR	NR	NR
Bilateral Ulnar	Wrist-ADM	NR	NR	NR	NR
Left Tibial	Ankle-Abd hal	4.00 (<4.80)	10.3 (>4.0)	NR	NR
Right Tibial	Ankle-Abd hal	3.85 (<4.80)	7.1 (>4.0)	NR	NR
Bilateral Peroneal	Ankle-EDB	NR	NR	NR	NR
	Fib. head-Tib. ant	NR	NR	NR	NR
Sensory nerve					
Left Median	Digit I-Wrist	2.10 (<3.70)	17.0 ↓ (>24.7)	53.3 (>48.1)	112
	Digit III-Wrist	2.94 (<3.70)	7.0 ↓ (>11.3)	54.4 (>49.5)	160
Right Median	Digit I-Wrist	1.77 (<3.70)	21.9 ↓ (>24.7)	53.1 (>48.1)	94
	Digit III-Wrist	2.40 (<3.70)	8.6 ↓ (>11.3)	55.8 (>49.5)	134
Left Ulnar	Digit V-Wrist	2.29 (<2.90)	3.8 ↓ (>7.2)	48.0 (>47.4)	110
Right Ulnar	Digit V-Wrist	2.16 (<2.90)	7.7 (>7.2)	51.9 (>47.4)	112
Left Tibial	Digit I-Ankle	4.20 (<6.10)	1.57 (>0.9)	47.1 (>35.1)	198
Left Peroneal	Calf-Fibular Head	5.10 (-)	1.89 (>1.20)	57.8 (>46.2)	295

↓, measured value was below the lower limit of the normal reference range; NR, no response; APB, abductor pollicis brevis; ADM, abductor digiti minimi; Abd hal, abductor hallucis; EDB, extensor digitorum brevis; Fib. head-Tib. Ant, Fibular head-Tibialis anterior. (-): There is no universally accepted normal reference range for the latency of peroneal sensory nerve conduction (calf to fibular head) due to significant anatomical variation. Values were presented as measured values (normal reference range). All reference ranges are from the neurophysiology laboratory of our institution.

## Data Availability

The data that support this study are available from the corresponding author upon reasonable request. Public sharing of these data is restricted to protect the privacy and confidentiality of the patient.

## Abbreviations

AHP	acute hepatic porphyrias
AIP	acute intermittent porphyria
HMBS	Hydroxymethylbilane synthase
ALA	δ-aminolevulinic acid
PBG	porphobilinogen
GBS	Guillain–Barré syndrome
IVIG	intravenous immunoglobulin
CT	computed tomography
ALT	alanine aminotransferase
CSF	cerebrospinal fluid
MRI	magnetic resonance imaging
EMG	electromyography
EICU	emergency intensive care unit
SWI	susceptibility-weighted imaging
FVC	F-wave conduction velocities
SIADH	syndrome of inappropriate antidiuretic hormone secretion
HGB	hemoglobin
5-HT	5-hydroxytryptamine
ALAS1	δ-aminolevulinic acid synthase 1
PGC-1α	peroxisome proliferator-activated receptor gamma coactivator 1-α
CMAPs	compound muscle action potentials
SNAP	sensory nerve action potential
siRNA	small interfering ribonucleic acid
